# Characterization of an Aptamer Targeting Neu5Gc, as an Endogenous Pathogenic Factor Derived from Red Meat

**DOI:** 10.3390/molecules29061273

**Published:** 2024-03-13

**Authors:** Yuxi Guo, Honglin Ren, Han Wang, Yiran Xiao, Cong Wang, Mengdi Liu, Fuchun Duan, Haosong Li, Pan Hu, Yansong Li, Zengshan Liu, Shiying Lu

**Affiliations:** State Key Laboratory for Diagnosis and Treatment of Severe Zoonotic Infectious Diseases, Key Laboratory for Zoonosis Research of the Ministry of Education, Institute of Zoonosis, College of Veterinary Medicine, Jilin University, Changchun 130062, China; yxguo21@mails.jlu.edu.cn (Y.G.); renhl@jlu.edu.cn (H.R.); wang_h19@mails.jlu.edu.cn (H.W.); xiaoyr20@mails.jlu.edu.cn (Y.X.); congwang20@mails.jlu.edu.cn (C.W.); liumd22@mails.jlu.edu.cn (M.L.); duanfc22@mails.jlu.edu.cn (F.D.); lhs948892860@outlook.com (H.L.); hupan84@163.com (P.H.); l_ys92305@163.com (Y.L.); zsliu1959@16-3.com (Z.L.)

**Keywords:** N-glycolylneuraminic acid (Neu5Gc), aptamer, enzyme-linked aptamer assay, red meat analysis

## Abstract

N-glycolylneuraminic acid (Neu5Gc), a sialic acid predominantly found in the non-neurohumoral fluids of hind-mouthed animals, is incapable of synthesizing Neu5Gc due to a deletion in the CMAH exon of the gene encoding human CMP-Neu5Gc hydroxylase. But consumption of animal-derived foods that contain Neu5Gc, such as red meat, can instigate an immune response in humans, as Neu5Gc is recognized as a foreign substance by the human immune system. This recognition leads to the production of anti-Neu5Gc antibodies, subsequently resulting in chronic inflammation. When Neu5Gc is consumed excessively or frequently, it may contribute to the development of heart disease and cancer. This makes Neu5Gc, an endogenous pathogenic factor derived from red meat, a new hot topic in red meat safety research. In this study, aptamers obtained by the magnetic bead SELEX technique were subjected to homology and secondary structure prediction analysis as well as affinity determination. The result indicated that the aptamer 2B.N2A9 exhibited a robust binding affinity, with an affinity constant (Ka) of 1.87 × 10^8^ L/mol. This aptamer demonstrated optimal binding specificity within a pH range of 5.4 to 7.4. Molecular docking analysis further revealed that aptamer 2B.N2A9 formed stable binding interactions with the target Neu5Gc at specific sites, namely G-14, C-15, G-13, G-58, G-60, and C-59. An Enzyme-Linked Oligonucleotide Sorbent Assay (ELOSA) methodology was established to detect the endogenous pathogenic factor Neu5Gc present in red meat. This method demonstrated a limit of detection (LOD) of 0.71 ng/mL, along with an average recovery rate of 92.23%. The aptamer obtained in this study exhibited favorable binding properties to Neu5Gc. The assay was relatively convenient and demonstrated good sensitivity. Further investigation into the distribution of Neu5Gc in various red meats is of public health significance and scientific potential. A practical detection method should be provided to guide red meat diets and ensure the nutrition and safety of meat products.

## 1. Introduction

The acidic N-glycolylneuraminic (Neu5Gc), as a major form of salivary acid, possesses a pyranose structure and consists of nine carbon atoms [1]. Sialic acid, primarily located at the termini of cell surface glycoconjugates in organisms, plays a crucial role in intercellular recognition, adhesion, inflammatory responses, as well as tumor cell growth and metastasis. The presence of Neu5Gc has been observed in the non-neural tissues and body fluids of most postoral animals, such as vertebrates and echinoderms [2], while it is absent in normal human tissues. The human CMAH gene lacks an exon, resulting in a premature truncation of the open reading frame and subsequent inactivation of CMP-Neu5Gc hydroxylase, leading to the body’s inability to synthesize Neu5Gc [3]. Although the body does not synthesize Neu5Gc endogenously, it can be obtained exogenously through the consumption of foods that are rich in Neu5Gc, such as red meat and milk [4]. The exogenous Neu5Gc enters the body and accumulates, triggering an antigen-antibody reaction that subsequently stimulates carcinogenesis by the promotion of chronic inflammation. Therefore, excessive or prolonged uninterrupted consumption of red meat may potentially contribute to the development of cardiovascular disease and cancer [5,6,7]. The International Agency for Research on Cancer (IARC) of the World Health Organization (WHO) released a report in 2015, classifying red meat such as beef, mutton, and pork as a Group 2A carcinogen. Therefore, ensuring effective detection of Neu5Gc is crucial for animal food safety.

The current detection methods for Neu5Gc primarily rely on liquid-phase instruments, which exhibit enhanced sensitivity in detecting glycoproteins and glycolipids containing Neu5Gc. Alternatively, acid-base hydrolysis can be employed to detect free Neu5Gc [8]. However, the costly instrumentation, specialized operation, and hydrolysis process of the samples exert some influence on the determination of content, thereby limiting the widespread application of this method. Immunological assays rely on Western blot techniques utilizing mono-(poly-)clonal antibodies against ganglioside GM2 and GM3, as well as immunohistochemistry for detecting glycolipid-containing Neu5Gc in certain tumor tissues. The specificity issue of antibodies, however, results in reduced specificity and increased background reactivity commonly observed in frequently used chicken polyclonal antibodies. The existing detection methods are insufficient to meet the demands of research and practical applications, primarily focusing on the identification of Neu5Gc-containing complexes. Nowadays, there is a limited project of research on Neu5Gc, a potentially hazardous substance found in red meat, with numerous mechanisms that remain incompletely understood and research methodologies that have yet to reach maturity. In particular, the lagging detection method hinders the advancement of in-depth research on Neu5Gc.

These days, nucleic acid aptamers, also known as “chemical antibodies” [9], represent a highly promising alternative recognition element for analysis. Nucleic acid aptamers are single-stranded DNA or RNA molecules isolated through SELEX (systematic evolution of ligands by exponential enrichment) screening that adopt stable secondary or tertiary structures, such as hairpins, stem loops, convex loops, and G-quadruplexes. These structures are formed through intermolecular forces such as van der Waals interactions, hydrogen bonding, electrostatic interactions, stacking of planar moieties, and complementary folding of shapes [10]. The discovery of aptamers in vitro in 1990 led to subsequent investigations into their applications. Aptamers exhibit comparable affinity and specificity to monoclonal antibodies, while also providing distinct advantages over antibodies: (i) sequences are confirmed through in vitro screening, thereby eliminating the potential risk of immunogenicity and toxicity; (ii) they exhibit stability and reduced susceptibility to environmental factors due to chemical synthesis and labeling; and (iii) they are easily obtainable with a short preparation cycle, low cost, and minimal batch-to-batch variation. The inherent characteristics of nucleic acid aptamers make them suitable for analytical, diagnostic, and therapeutic applications [11]. An aptasensor based on indirect competition assay has been successfully developed by Sai Wang et al. [12]. And the use of a biotin-enzyme-labeled affinity amplification system, constructed through indirect competition, was reported for detecting tetracycline. The preferred aptamer was used as the detection molecule and solid-phase antigen competition was adopted as the target for this package. The sensor achieved a limit of detection (LOD) of 9.6 × 10^−3^ ng/mL and had a wide linear range of 0.01–100 ng/mL. Khalil Abnous et al. developed a novel colorimetric sandwich aptasensor specifically designed for the detection of chloramphenicol, utilizing an indirect competitive enzyme-free method. This innovative approach employed a 40 mer ssDNA aptamer as a targeting agent and achieves an impressive limit of detection (LOD) as low as 451 pM [13]. Our research group has previously introduced the Neu5Gc-binding aptamer, which was designed through SELEX, which addresses the limitation of animal source antibody preparation in the development of immunological detection technology [14]. Additionally, we have developed an aptamer-based immunochromatographic test strip [15]. Recently, ELISA-based aptasensors have been widely reported for their applications in detecting cancer cells, foodborne pathogens, and small molecules [16,17]. In this paper, we present a feasibility analysis of aptamer-based detection of Neu5Gc in red meat samples.

In this study, the aptamers were screened using the magnetic bead SELEX technique. Homology analysis and secondary structure prediction were employed for aptamer structural analysis. The screened aptamers were further subjected to affinity assay and the pH stability and specificity of the best aptamer were determined. An indirect competitive enzyme-linked aptamer assay was developed utilizing an 81mer-ssDNA (Ka = 1.87 × 10^8^ L/mol) for precise determination of Neu5Gc in red meat samples. The assay successfully established a standard curve, demonstrating excellent sensitivity (limit of detection, LOD = 0.71 ng/mL) and a broad linear range (1.29–69.2 ng/mL) performance on good, spiked recoveries. The molecular docking method was employed to identify the specific binding sites of aptamers to Neu5Gc. and to provide a solid theoretical foundation and technical support for ensuring the safety of red meat food. The development of a Neu5Gc assay based on aptamers not only solves the problem of difficult antibody preparation in existing antigen-antibody-based immunological techniques, but also promotes the development of traditional antigen-antibody-based immunological techniques and broadens the application fields of these techniques. Compared to the current high-performance liquid chromatography (HPLC) detection method, the newly established immunological rapid detection method significantly reduces the cost of detection. This method can be directly applied to investigate the correlation between the content level of this pathogenic factor and health and disease in red meat-related foods. It is beneficial for ensuring nutritional safety and promoting the health of red meat while demonstrating promising prospects for application.

## 2. Results

### 2.1. Production and Analysis of the Solid Phase Antigen Neu5Gc-BSA

**Figure 1:**
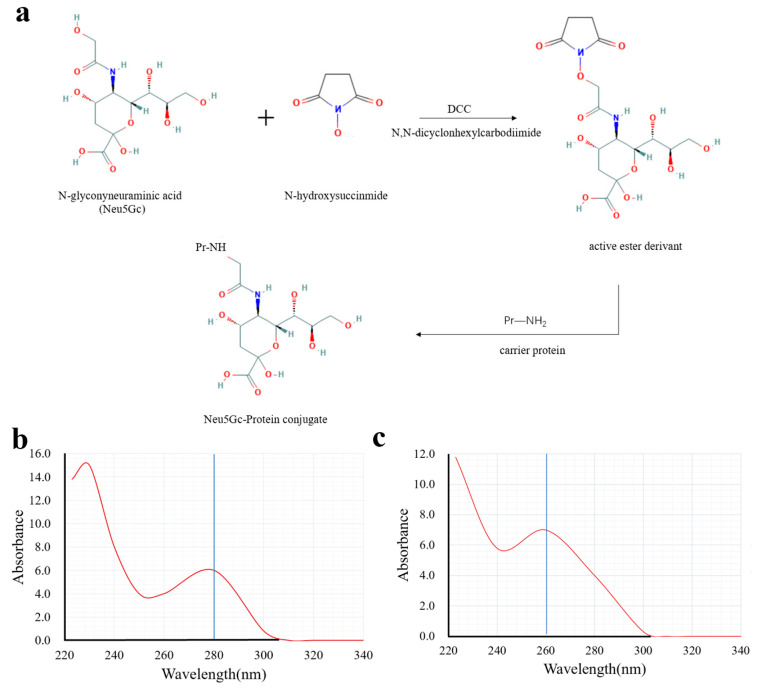
Binding reaction scheme and identification of Neu5Gc with carrier proteins. (**a**) Synthesis reaction scheme of Neu5Gc and BSA, (**b**) UV spectrum of BSA control, (**c**) UV spectrum of Neu5Gc-BSA coupling.

To address the difficulty of direct immobilization of Nu5Gc, this aptamer was coupled with BSA. As a result, we obtained the solid-phase antigen Neu5Gc-BSA by immobilizing Neu5Gc on the enzyme plate through the binding of BSA to the plate. Activated esters of Neu5Gc are usually prepared by the activated ester method and the carbodiimide method. In this study, carboxylic acid-based protein couplers of Neu5Gc were synthesized using the activated ester method. In the presence of DCC, the carboxyl group of Neu5Gc reacted with N-hydroxy succinimide to generate the affixes by reacting with the amino groups of carrier proteins (IgG and BSA). The reaction scheme is shown in Figure 1a. The Neu5Gc-BSA coupling was analyzed by UV spectroscopy. Different substances showed different UV absorption spectra and, the absorbance or absorption wavelength of the carrier protein BSA changed after coupling with Neu5Gc. As shown in Figure 1b,c, the absorption spectrum shifted from approximately 280 nm (Figure 1b) to 260 nm (Figure 1c) upon addition of the coupling agent. This observation confirms the successful completion of the coupling process.

### 2.2. Sequence Analysis of Nucleic Acid Aptamers

**Figure 2:**
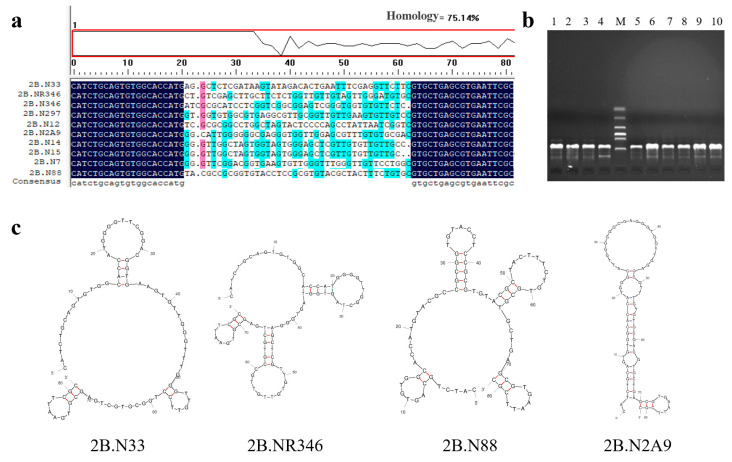
Sequence analysis of aptamers: (**a**) homology analysis of the screened aptamer; (**b**) the result of the selected ssDNA. M: DL 2000 Marker; 1–10 are the 10 candidate aptamers, respectively; (**c**) secondary structure prediction of candidate aptamer sequences.

Sequence homology was analyzed using DNAMAN 9.0 software, and the secondary structure of the sequence was predicted by m-fold (http://www.mfold.org/, accessed on 1 April 2022). The PCR products of the aptamer sequences were confirmed by 2% agarose gel electrophoresis, and the expected target product of 81 bp was obtained (Figure 2b). DNAMAN analysis revealed 75% homology of the sequences (Figure 2a). m-Fold predicted the thermodynamic parameter ΔG and the secondary structure of the aptamers. ΔG represents the Gibbs free energy for the formation of the secondary structure of the aptamer, and the higher the absolute value, the more stable the sequence. Most of the screened sequences have consecutive G-bases, forming a G-triple or G-quadruplex structure. This structural feature also enhances the stability of the nucleic acid sequences, which can be used as the key region and binding site for Neu5Gc small-molecule recognition by the nucleic acid aptamer. As shown in Figure 2c, the secondary structure of the aptamer exhibits various hairpin, stem-loop, and bulge structures, which can stack planar motifs and form hydrogen bonds that are involved in the formation of tertiary binding sites between the nucleic acid aptamer and the target molecule. After homology and secondary structure analysis, four nucleic acid aptamer sequences, 2B.33, 2B.NR346, 2B.88, and 2B.N2A9, were selected for subsequent experiments.

### 2.3. Affinity Measurement Results

**Figure 3:**
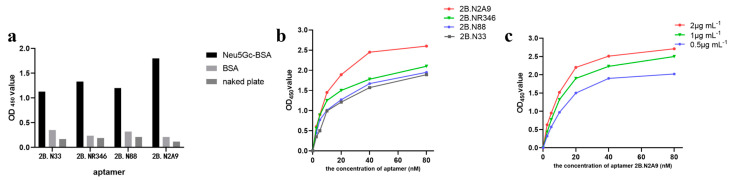
Affinity determination: (**a**) candidate aptamer specificity was determined by Neu5Gc-BSA and BSA plates, and the concentration of the aptamer in the test was 50 nM; (**b**) the affinity of the four aptamers was determined by indirect enzyme-linked immunosorbent assay with the aptamer concentration and OD_450_ values on the X and Y axes, respectively; (**c**) the affinity constant of 2B.N2A9 was determined by coating Neu5Gc-BSA at concentrations of 0.5, 1 and 2 μg mL^−1^.

To evaluate the specificity of the selected aptamers coated with Neu5Gc-BSA and BSA, 2B.33, 2B.NR346, 2B.88, and 2B.N2A9 showed, respectively, significant specificity for the target molecules, with 2B.N2A9 having the highest binding activity (Figure 3a). To further understand the binding strength of the four aptamers to the target, an indirect enzyme-linked immunosorbent assay was used to determine the affinity of the four aptamers being 2B.N2A9 the best (Figure 3b). The ability of aptamer 2B.N2A9 to encapsulate different concentrations of Neu5Gc-BSA was evaluated (Figure 3c). Affinity constants were calculated using the formula:

(1) $$Ka=\frac{\left(n-1\right)}{2({n[Ap\prime ]}_{t}-{\left[Ap\right]}_{t})}$$

where n is the ratio of the two Neu5Gc-BSA encapsulation concentrations in the same set (n > 1), and [Ap′]_t_ and [Ap]_t_ are the aptamer concentrations corresponding to 50% of the maximum OD_450_ of both curves ([Ap′]_t_ > [Ap]_t_). The mean value was taken as the affinity constant. The affinity constants of aptamers 2B.33, 2B.NR346, 2B.88, and 2B.N2A9 were 1.05 × 10^8^, 1.33 × 10^8^, 1.11 × 10^8^, and 1.87 × 10^8^ L/mol, respectively. It is noteworthy that among the four aptamers, the affinity of aptamer 2B.N2A9 for Neu5Gc was higher than that of the other aptamers.

### 2.4. Analysis of the Binding Mechanism

**Figure 4:**
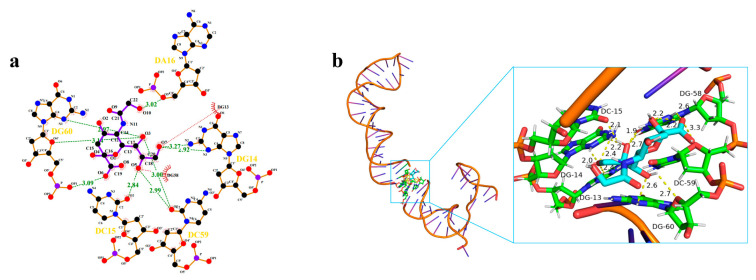
Molecular docking: (**a**) two-dimensional force analysis: in this two-dimensional diagram, the green dashed line indicates hydrogen bonding, and the red dashed line indicates hydrophobic forces; (**b**) the three-dimensional view of the interaction between Neu5Gc and 2B.N2A9 of aptamer. In the three-dimensional diagram, the yellow dashed line indicates hydrogen bonding.

The binding model of the 2B.N2A9 chimera with Neu5Gc was further analyzed using the molecular docking technique, and the interaction between the two was explored at the molecular level. The binding energy between Neu5Gc and the 2B.N2A9 aptamer was −7.0 kcal/mol, which allowed stable binding. As shown in the three-dimensional diagram (Figure 4b), the small Neu5Gc molecule can bind to the G-base 14 of the receptor single-stranded nucleic acid (2B.N2A9) via all three hydrogen bonds of 2.0 Å, 2.2 Å, and 2.4 Å, respectively; to the C-base 15 and G-base 13 of the receptor 2B.N2A9 via two hydrogen bonds of 2.1 Å and 2.4 Å, respectively; to C-base 15 of receptor 2B.N2A9 via five hydrogen bonds of 1.9 Å, 2.9 Å, and 2.4 Å, respectively; to C-base 13 of receptor 2B.N2A9 via five hydrogen bonds of 1.9 Å, 2.9 Å, 2.4 Å, respectively. 1.9 Å, 2.7 Å, 2.2 Å, 2.2 Å, 2.6 Å to G-base 58 of receptor 2B.N2A9; via two hydrogen bonds of 2.6 Å and 2.7 Å to G-base 60 of receptor 2B.N2A9; and via a hydrogen bond of 3.3 Å to C-base 59 of receptor 2B.N2A9. The major binding sites of aptamer 2B.N2A9 to Neu5Gc were G-14, C-15, G-13, G-58, G-60, and C-59. Two-dimensional interaction force analysis yielded similar results (Figure 4a).

### 2.5. pH Stability and Specificity Measurements

**Figure 5:**
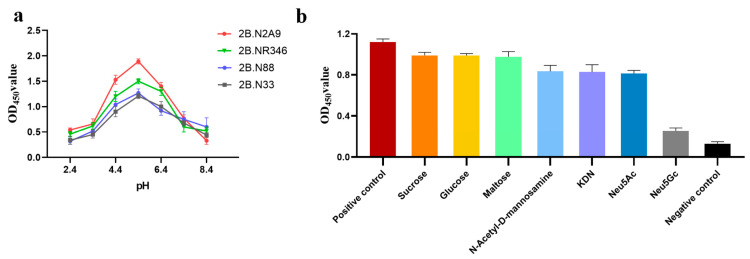
pH stability and specificity measurements: (**a**) effect of pH on Neu5Gc binding affinity; (**b**) the specificity of 2B.N2A9 aptamer candidates for Neu5Gc, and the analogs: Neu5Ac, KDN (keto-deoxy-nonanoic acid), N-acetyl-D-mannosamine, maltose, sucrose, and glucose. All the tests and experiments were repeated three times biologically. The data are shown as mean ± SD.

The three-dimensional conformation of nucleic acid aptamers is sensitive to environmental conditions within the system, and the microenvironment (e.g., pH) affects the structural integrity of the aptamer and its interaction with the target. To investigate the effect of pH on binding affinity, the binding of Neu5Gc to the aptamer under different pH conditions was detected by indirect ELISA. The results showed that optimal binding affinity was observed in the pH range of 5.4–7.4, while weaker binding affinity was observed in the lower and higher pH ranges. Compared to other nucleic acid aptamer candidates, 2B.N2A9 showed stronger binding activity in a specific pH range (Figure 5a). The specificity of these sequences for the target was further assessed using the same method, and as shown in Figure 5b, 2B.N2A9 was more specific for Neu5Gc. The results of the calculated cross-reactivity are shown in Table 1, with a cross-reactivity of 2.0% between Neu5Gc and Neu5Ac. There was no significant cross-reactivity between Neu5Gc and the other analogues, suggesting a better specificity of the method. Therefore, aptamer 2B.N2A9 was determined to be the best choice for Neu5Gc detection based on its binding affinity, specificity, and pH stability.

### 2.6. Aptamer-Based ELOSA Assay Feasibility Analysis

The Neu5Gc standards were used as competitors for the ELOSA at mass concentrations of 0, 0.78125, 1.5625, 3.125, 6.25, 12.5, 25, 50, 100, 200, and 400 ng/mL. To ensure accuracy and precision in the results obtained from these experiments, three parallel experiments were conducted for each concentration with a standard deviation within a range of ±10%. The standard curve was constructed using the common logarithm of the standard concentration as the x-axis and (B/B_0_) as the y-axis, where B represents the OD_450_ value corresponding to different concentrations of Neu5Gc in the standard wells, and B_0_ represents the OD_450_ value corresponding to zero Neu5Gc in the standard wells. The detection range was determined based on an inhibition of 20–80%. The linear regression equation was y = −0.3417x + 0.837 (R^2^ = 0.9927), while the detection range spanned from 1.29 to 69.2 ng/mL. The limit of detection (LOD) was determined [18].

(2) $$LOD=3\times \frac{SD}{S}$$

SD represents the standard deviation of the blank value without Neu5Gc competition and S denotes the slope of the established standard curve. LOD was determined to be 0.71 ng/mL using the equation, representing concentration.

### 2.7. Feasibility Analysis of Aptamers in the Detection of Red Meat Samples

To verify the practical applicability of the aptamer-based method, Neu5Gc was detected in real samples. As shown in Table 2, the total Neu5Gc content was determined in different types of red meat. The results showed that the highest Neu5Gc content was found in beef (23.60 ± 1.21) μg/g, followed by pork (17.67 ± 1.03) μg/g and mutton (15.33 ± 0.66) μg/g. Next, different concentrations of Neu5Gc standards were spiked into the red meat samples to determine the recoveries of the free state using the formula.

The recoveries of beef samples ranged from 87% to 103.94%; the recoveries of mutton samples ranged from 86.3% to 97.52%; and the recoveries of pork samples ranged from 82% to 107.32%, which verified that the method was able to accurately detect the red meat samples with high recoveries and precision. Meanwhile, the spiked recoveries were validated by HPLC, and the recoveries of beef, pork, and mutton samples were 100.41%, 98.80%, and 101.49%, which were similar to those of 10 ng/mL. This indicates that the selected aptamers can be used to establish the assay and then be used to quantitatively analyze Neu5Gc in real samples.

AOAC requires that the recovery be in the range of 50~150% [19]. Too low or too high requires modification of the experimental protocol or adjustment of the linear range of the standard curve. In this experiment, red meat samples were ground using a mortar and pestle, and then Neu5Gc was extracted with NaOH and acetic acid [20], and the average recoveries obtained were 92.23%, which indicated that the selected sample treatment, the type and concentration of extractant, and the sample matrix did not cause any significant interference in the assay, and the method has the value of application for the detection of real samples.

## 3. Discussion

Nowadays, food safety is receiving increasing attention. According to epidemiological studies, long-term consumption of red and processed meats is one of the risk factors for cancer in humans. Red meat was classified as a class 2A human carcinogen by the World Health Organization (WHO) in 2015. Diets high in red meat are estimated to contribute to approximately 50,000 cancer-related deaths worldwide annually. Although there have been numerous attempts to explain the association, a clear causal relationship has not been demonstrated. It is worth noting that Neu5Gc is more specific to red meat than other possible causative agents in red meat. Additionally, Neu5Gc is the major type of salivary acid in most mammals and is highly abundant in red meat, but rare in poultry and fish. The inability to synthesize Neu5Gc in humans is prevented by the deletion of exon CMAH of the gene encoding human CMP-Neu5Gc hydroxylase [21]. However, consumption of animal-derived foods such as red meat [22] and milk containing Neu5Gc can lead to its recognition as a foreign substance by the human immune system and subsequent production of anti-Neu5Gc antibodies [23], resulting in chronic inflammation [24]. The presence of Neu5Gc antibodies can lead to chronic inflammation, potentially resulting in heart disease and cancer when consumed excessively or regularly [25,26,27]. Therefore, it is important to ensure the level of Neu5Gc in red meat for food safety [28].

Traditional mass spectrometry techniques for Neu5Gc detection rely on sophisticated instrumentation and incur high costs [29], rendering them unsuitable for underdeveloped countries or regions with limited resources [30]. Immunological detection techniques, on the other hand, are constrained by the challenges associated with generating animal-derived antibodies and primarily focus on identifying Neu5Gc-containing complexes, with Table 3 providing a summary of these methods along with their respective characteristics. These methods are often time-consuming, dependent on expensive instrumentation, and involve complex laboratory operations. There is a need for low-cost, sensitive nucleic acid assays that do not require specialized auxiliary equipment. Aptamers known as “chemical antibodies” as novel probes have unique advantages in substance detection and are favored by many researchers for their rapid synthesis and easy application. In vitro, SELEX enables the direct isolation of aptamers for virtually any small molecule, including non-immunogenic or toxic ones. Aptamer sensors for small molecule detection [31,32,33] also offer new options for Neu5Gc detection. The successful screening of nucleic acids involves adaptive folding into specific spatial structures in the screening buffer, enabling them to bind specifically to the target. Consequently, this process typically requires a specific temperature environment and ion regime. The optimal pH of 2B.N2A9 binding buffer was determined to ensure optimal binding of the aptamer to the target. The assay in this study combines the nucleic acid aptamer 2B.N2A9 as a recognition element with an enzyme-linked amplification reaction, wherein an enzyme molecule is introduced into a bioanalytical method to achieve signal amplification through its catalytic effect. The most commonly employed technique is ELISA, wherein the antibody facilitates the capture of the target molecule. Subsequently, an enzyme conjugated with a secondary antibody is introduced, catalyzing a substrate and generating a color reaction, thereby achieving signal amplification. The utilization of nucleic acid aptamers for the detection of antibiotics in food is limited. Furthermore, there is a scarcity of research on combining nucleic aptamers with enzyme-linked amplification reactions for the quantitative detection of things like Neu5Gc. The sensor developed in this study offers a more convenient method for detecting Neu5Gc, which can be detected routinely in batches. This resolves the issue of the limited development of immunological detection technology due to the difficulty of preparing animal-derived antibodies. Furthermore, this assay has the added benefit of lower sensitivity and not requiring complex instrumentation.

There have been several reports on the detection methods of salivary acid. Marefa Jahan et al. [42,43] used UHPLC to measure Neu5Gc, Neu5Ac, and KDN levels in skeletal muscle and organ tissues from several species. Shewell et al. [44] utilized the SubB2M-SPR assay to investigate Neu5Gc-containing glycoconjugates in the serum of patients with cutaneous melanoma. Additionally, the team developed an enhanced SubB2M-based SPR assay [45] and employed it to examine serum samples collected from patients with breast cancer to determine any association between the detection of the Neu5Gc biomarker and breast cancer screening and surveillance. Jieun Kim et al. [46] identified and quantified salivating N-glycans using ultra-performance liquid chromatography (UPLC) and liquid chromatography (LC), electrospray ionization (ESI), high-energy collisional dissociation (HCD), and tandem mass spectrometry (MS/MS). And based on composite materials to construct biosensors for the relevant detection of salivary acid analogs [47,48]. The study showed that the above detection methods have a long preparation time and detection cycle. In contrast, the Elisa method using nucleic acid aptamers does not require a lot of preparation work, and the detection cycle is only 2–3 h, which is 20–30 times shorter than the traditional method and has the same performance in terms of sensitivity. It was reported that Hui et al. [49] carried out the detection of Neu5Ac based on nucleic acid aptamer and compared the binding efficiency of the screened aptamers to Neu5Ac by three different SELEX screening methods. In the present study, the molecular recognition mechanisms of 2B.N2A9 and Neu5Gc are explored by molecular docking [50], the binding sites of target and aptamer are identified, and the excellent binding strengths of both are further verified [51]. In conclusion, we have successfully identified and characterized an aptamer specifically targeting Neu5Gc through a comprehensive screening and analysis process. We have evaluated the feasibility and practicability of detecting Neu5Gc in red meat samples. Varki [52] studied the levels of Neu5Gc in pork and beef and found that they were 30.1 µg/g in beef and 25.5 µg/g in pork. Chen et al. [53] reported that Neu5Gc was 5.6 µg/g in pork and 30.3 µg/g in beef. In China, Jiang Yun et al. [54] found that (35.00 ± 2.41) µg/g of Neu5Gc was present in pork and (19.00 ± 1.25) µg/g in beef. The results of the present study were consistent with the above findings. There are three possible reasons for the differences in the results. First, there may be differences between sampling points. Second, the same animal may differ in breed and feeding method, and the Neu5Gc content may also be affected by different breeds and different feeding methods. Lastly, different sample pretreatment methods may also lead to different test results. Therefore, this innovative assay based on aptamers has great potential to detect Neu5Gc in red meat.

## 4. Materials and Methods

### 4.1. Materials

The nucleic acid sequences utilized in this study, as presented in Table 4, were synthesized and purified via high-performance liquid chromatography (HPLC) by Sangon Biotech (Shanghai, China). Neu5Gc and KDN were obtained from Shanghai Cosun Technology Co. The Neu5Ac was obtained from TCI Co. Ltd. (Shanghai, China). D-(+)-glucose, sucrose, and D-(+)-maltose monohydrate were obtained from Sinopharm Chemical Reagent Corporation (Shanghai, China). Bovine serum albumin (BSA), horseradish peroxidase-labeled streptavidin (SA-HRP), and 3,3′,5,5′-tetramethylbenzidine dihydrochloride (TMB) were obtained from KPL (Gaithersburg, MD, USA). Double-distilled water was utilized in the experiments. The reagents utilized for the synthesis were all of analytical grade and purity, sourced from China Pharmaceutical Group Corporation (Shijiazhuang, China), thereby obviating the need for additional purification. The transparent 96-well enzyme labeling plates were obtained from JITE Biofiltration Co. Ltd. (Guangdong, China); and the multifunctional enzyme labeling instrument (cytation-5) was acquired from Biotek Biologicals, located in Winooski, VT, USA; the thermostatic incubator (DHP-120) was acquired from the Experimental Instrumentation Factory located in Shanghai, China; the Centrify (ST16R) and rotary evaporator (RE-3000A) were acquired from Physical and Chemical Equipment’s Inc. (Tokyo, Japan).

### 4.2. Preparation of Neu5Gc-BSA Conjugate

Neu5Gc, as a small molecule, lacks immunogenicity. The active ester method was employed in this study to couple Neu5Gc with the carrier protein BSA, resulting in the preparation of an experimental target encapsulant. The carboxyl group of Neu5Gc was utilized in conjunction with N-hydroxy succinimide to generate an active ester in the presence of DCC (*N*,*N*′-dicyclohexylcarbodiimide). This active ester then reacts with the amino group of BSA, leading to a coupling between the carrier and small molecule for a solid-phase antigen.

### 4.3. The Acquisition of Nucleic Acid Aptamers and Analysis of Predictions

**Figure 6:**
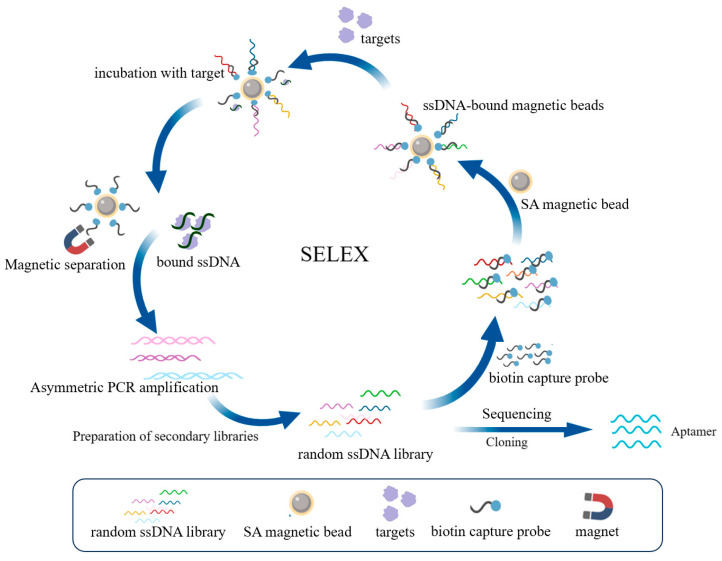
Selection procedure of ssDNA library immobilized magnetic beads SELEX technique.

The SELEX screening procedure is shown in Figure 6 [55]. Neu5Gc was used as the screening target using ssDNA library immobilized magnetic bead SELEX technology. Sequences binding to Neu5Gc were enriched during the screening process, and the PCR products were cloned and sequenced by Sangon Biotech (Shanghai, China) Co. The homology of the sequences was analyzed using DNAMAN software, and the secondary structure of the sequences was predicted by M-fold (http://www.mfold.org/, accessed on 1 April 2022).

### 4.4. Determination of Binding Affinity for Candidate Aptamers

The affinity of the aptamer was determined by ABS-ELISA. Neu5Gc-BSA coupling was diluted to 1 μg/mL in 0.1 M carbonate buffer (pH 9.6), immobilized on a microtiter plate, and incubated overnight at 4 °C. Then, skimmed milk closure. The aptamer was diluted to a concentration of 1:200 with SHCMK binding buffer. It was bound to the Neu5Gc-BSA target on the plate surface and incubated at 37 °C for 1 h. Enzyme-conjugated streptavidin was added and incubated for 1 h at 37 °C. TMB substrate was then added, and the reaction was stopped with 2M H_2_SO_4_ for 10 min. The OD value at 450 nm was measured using enzyme labeling [12].

### 4.5. Molecular Docking Methods

Molecular docking studies were performed using Autodocking Vina (version 1.1.2) software, which provides theoretical support for studying the binding sites of nucleic acid aptamers to Neu5Gc. First, single-stranded modeling of nucleic acid (DNA) sequences was performed using the 3DRNA server to dock nucleic acids to small molecules. Nucleic acids were designated as receptors and small molecules as ligands. Autodock Tools (http://mgltools.scripps.edu/downloads, accessed on 1 April 2022) was used to hydrogenate, check and calculate charges, designate atom types as AD4 types, and construct docking grid boxes for nucleic acid structures. Finally, both the nucleic acid structure format and the small-molecule ligand format were to be converted from PDB to PDBQT in Autodock Tools for further docking. After docking using Vina, the scores of the two-by-two combinations of nucleic acids and small molecules were calculated, the forces were analyzed and visualized in three-dimensional angles using Pymol software (version 2.2.0), and a two-dimensional map of the interactions between small molecules and nucleic acids was constructed using Ligplus software, Version 2.2 [50].

### 4.6. The Evaluation of pH Stability and Specificity of Aptamers

To evaluate the effect of pH on binding affinity, sequence 2B.N2A9 was incubated at different pH conditions (4.4, 5.4, 6.4, 7.4, 8.4, and 9.4) using an indirect ELISA with the same experimental steps as in 4.4. All experiments were performed in triplicate, and pH stability was evaluated separately at different pH conditions.

Based on affinity analysis, the aptamer 2B.N2A9 was used to assess specificity. Cross-reactivity was determined with structural analogues of Neu5Gc, including Neu5Ac, KDN (ketodeoxynonanoic acid), N-acetyl-D-mannosamine, maltose, sucrose, and glucose at 50 ng/mL concentration, using Neu5Gc standard solution as a control. All experiments were performed in triplicate. Specificity analysis was performed by calculating the cross-reactivity (CR) [56]:

(3) $$CR(\%)=\frac{{IC}_{50}(Neu5Gc)}{{IC}_{50}\left(Other\;structural\;analogues\right)}$$

### 4.7. Establishment of an ELOSA Assay for Neu5Gc Based on Selected Aptamer

**Figure 7:**
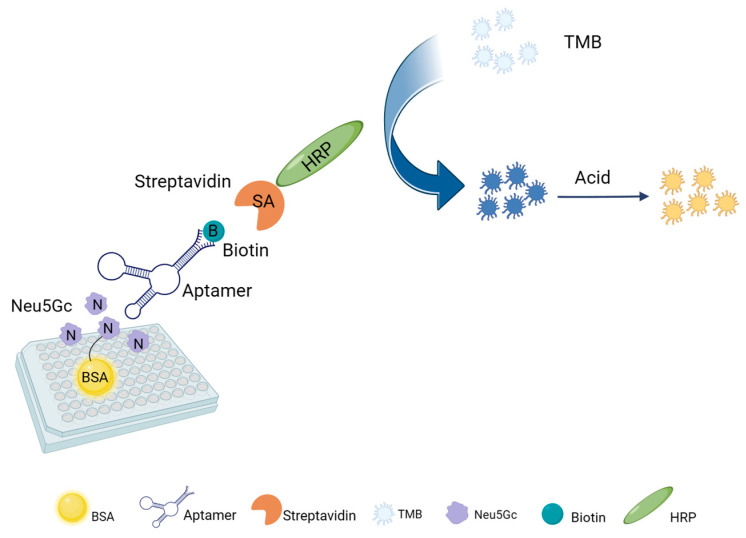
Scheme of ELOSA. Competition between immobilized Neu5Gc–BSA and free Neu5Gc in standard or red meat solution for the biotinylated aptamer, incubation with the Streptavidin–HRP conjugate, and subsequent enzyme label detection.

An aptamer-based ELOSA method for Neu5Gc detection was further established to demonstrate the potential application of the selected aptamer. The standard operating procedure (SOP) is shown in Figure 7. Neu5Gc-BSA coupling was encapsulated at the bottom of the enzyme plate, and the excess sites of the plate were sealed with a sealer, after which the sample and the aptamer 2B.N2A9 were added. The immobilized Neu5Gc would compete with the Neu5Gc in the sample for a limited number of nucleic acid aptamers. After competition, the mixed solution in the enzyme plate was washed to remove the aptamer, which was combined with the Neu5Gc-BSA-bound aptamer 2B.N2A9 to the bottom of the enzyme plate, and the biotin-labelled aptamer 2B.N2A9 then specifically binds to SA-HRP to introduce the enzyme molecule into the system. Finally, HRP catalyzes the color reaction of the substrate, TMB. The target compounds were quantitatively detected by the change in absorbance (OD_450_) of the colored substrate. The absorbance (OD_450_) of each well was measured using an enzyme-linked immunosorbent assay, and a higher OD_450_ value indicated a lower level of free Neu5Gc in the sample. After optimization of each condition, the best response was determined as follows: encapsulation concentration of 1μg/mL, 5% skimmed milk powder incubated at 37 °C for 2 h, optimal working concentration of primary antibody adjusted to 50 nmol/L, incubation at 37 °C for 1 h, secondary antibody and incubation at 37 °C for 1 h, and optimal working time of substrate was 10 min.

### 4.8. Preparation and Analysis of Red Meat Samples

The feasibility of consuming the tenderloin portions of beef, pork, and mutton was tested due to their frequent consumption. Red meat samples were obtained from a local market in Jilin Province, China. Neu5Gc was first extracted from the red meat samples [57]. 0.1 g of red meat samples were weighed, sprayed uniformly with 1, 10, and 50 ng/mL of Neu5Gc standards using a syringe, ground, and used to determine the spiked recoveries. Then 0.9 mL of NaOH (0.1 M) solution was added; the samples were homogenized and incubated at 37 °C for 30 min before neutralization with hydrochloric acid; 0.3 mL of the neutralized homogenate was taken; 0.3 mL of acetic acid (4 M) was added; and the sample was incubated at 80 °C for 3 h. The sample was cooled to room temperature and centrifuged at 14,000 rcf for 10 min. The supernatant was filtered through a 0.22 μM membrane to obtain the filtrate as total Neu5Gc; 0.2 mL of the neutralized homogenate was taken and 0.2 mL of acetic acid (4 M) was added, and centrifuged at 14,000 rcf for 10 min. The supernatant was filtered through 0.22 μM filter membrane to obtain the filtrate as free Neu5Gc. To verify the practicality of the method, the Neu5Gc content of the actual red meat samples was determined. The extracted Neu5Gc from red meat was detected using the ELOSA established above. The experiment was repeated three times in parallel [30], the actual samples were tested, and the spiked recoveries were calculated according to Equation (4).

(4) $$Spiked\;Recovery\;Rate=\frac{Spiked\;Result\;Detected\;After\;Spiking-Result\;Detected\;Without\;Spiking}{Actual\;Spiked\;Amount}\times 100\%$$

## 5. Conclusions

In this study, the homology analysis and secondary structure prediction of the selected aptamers were conducted using the magnetic bead SELEX technique. The affinity of the four predicted aptamers was determined through the indirect ELISA method. Among them, 2B.N2A9 exhibited exceptional affinity for Neu5Gc, with a Ka value of 1.87 × 10^8^ L/mol. Furthermore, within the pH range of 5.4–7.4, 2B.N2A9 demonstrated remarkable specificity and pH stability towards Neu5Gc. G-14, C-15, G-13, G-58, G-60, and C-59 are the binding sites between aptamer 2B.N2A9 and target Neu5Gc. A standard curve (y = −0.341x + 0.837; R^2^ = 0.9927) was established using the selected aptamers with a detection limit of 0.71 ng/mL and an average recovery rate of 92.23% and validated by HPLC. An aptamer-based ELOSA assay was developed to detect the endogenous hazardous factor Neu5Gc in red meat without the need for complex instrumentation. Inadequate, the sequence length of the nucleic acid aptamer can be optimized to improve the utilization of the aptamer. A more comprehensive and systematic comparative analysis of Neu5Gc levels in red meat is lacking. A prospective understanding of the Neu5Gc content levels in individual red meats is important to rationally guide people’s diets and safeguard dietary health. This method was used to further evaluate the ability to detect Neu5Gc in actual samples as well as to understand the distribution level of Neu5Gc content in red meat-related products, which provides data support for a rational diet.

## Figures and Tables

**Table 1:** The cross-reactivity of this ELOSA.

Neu5Gc and Its Analogues	Structural Formula	IC_50_ (ng/mL)	Cross-Reaction Rate (%)
Neu5Gc	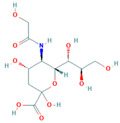	9.35	100.0%
Neu5Ac	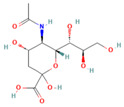	467.74	2.0%
KDN	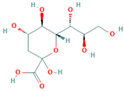	1659.58	0.56%
N-acetyl-D-mannosamine	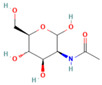	36,307.8	0.026%
Maltose	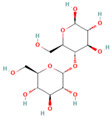	>93,500.00	<0.01%
Sucrose	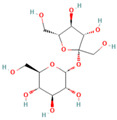	>93,500.00	<0.01%
Glucose	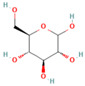	>93,500.00	<0.01%

**Table 2:** Actual samples testing.

Sample	Free of Neu5Gc (µg/g, Mean ± SD) (n = 3)	Conjugated of Neu5Gc (µg/g, Mean ± SD) (n = 3)	Total of Neu5Gc (µg/g, Mean ± SD) (n = 3)
Beef–Steak	2.22 ± 0.07	21.38 ± 1.04	23.60 ± 1.21
Pork–Griskin	1.217 ± 0.05	16.45 ± 1.01	17.67 ± 1.03
Mutton–Tenderloin	1.379 ± 0.22	13.95 ± 0.46	15.33 ± 0.66

**Table 3:** Overview of methods to detect Neu5Gc.

Method	Methodological Features	Reference
Immunohistochemistry	Polyclonal anti-Neu5Gc chicken IgY was used as an immunogen to recognize gangliosides with the Neu5Gc α2-R terminus. It was applied to immunohistochemistry and TLC overlay.	[34]
HPAE-PAD	The sialic acid content in glycolic acid hydrolysate was determined using high-performance anion exchange chromatography with pulsed amperometric detection (HPAE-PAD). Additionally, the separation of Neu5Ac and Neu5Gc in glycolic acid hydrolysate was achieved on an anion exchange column.	[35,36]
Mass spectrometry	The signal-to-noise ratio of RP-HPLC tandem MS interfaced with an electrospray ionization source (ESI) for the purification of released Neu5Gc in mass spectrometry and selective ion monitoring can be improved by using its isotopes as internal standards and avoiding derivatization.	[37]
Fluorescent marker	The free Neu5Gc was detected through fluorescence labeling with 1,2-diamino-4,5-methylenedioxybenzene (DMB) and subsequent RP-HPLC analysis of the derivative.	[38]
Mass Spectrometry (MS)	To facilitate purification and enhance the signal in MAL-DI-TOF mass spectrometry, it is necessary to detect Neu5Gc-carrying glycans by mass spectrometry, permethylate released N- or O-glycan mixtures, and destroy the O-acetyl group through alkaline permethylation for specific release of the glycans.	[39,40,41]
Aptamer detection	The Systematic Evolution of Ligands by Exponential Enrichment (SELEX) can be used to select aptamers from chemically synthesized nucleic acid libraries. This method can provide a new analytical probe for developing biosensors to detect Neu5Gc in animal-derived food as well as in the tissues and sera of tumor patients.	[14]

**Table 4:** Sequences of oligonucleotides used in this study. 5′-CATCTGCAGTGTGGCACCATG-N40-CGTGCTGAGCGTGAATTCGC-3′.

Sequence Name	Sequences (5′–3′)	Product Length
2B.N33	CATCTGCAGTGTGGCACCATGGGGTTCGGACGGTGAAGTGTTGGGTTTGGGTTGTTCCTGGCGTGCTGAGCGTGAATTCGC	81 bp
2B.NR346	CATCTGCAGTGTGGCACCATGGGGTTGGCTAGTGGTAGTGGGAGCTCGTTGTGTTGTTGCCGTGCTGAGCGTGAATTCGC	80 bp
2B.N88	CATCTGCAGTGTGGCACCATGTACGCCGCGGTGTACCTCCGCGTGTACGCTACTTTCTGTGCGTGCTGAGCGTGAATTCGC	81 bp
2B.N2A9	CATCTGCAGTGTGGCACCATGGGCATTGGGGGGCGAGGGTGGTTGGAGCGTTTGTGTGCGACGTGCTGAGCGTGAATTCGC	81 bp

Remarks: 5′ end-modified biotin.

## Data Availability

Data is contained within the article.
